# How to form shared objects to enhance university–school collaboration? A cultural–historical activity theory perspective

**DOI:** 10.3389/fpsyg.2024.1307552

**Published:** 2024-03-27

**Authors:** Xueqin Fang, Qiming Mao, Jianzhong Janne Hong, Chunting Diao

**Affiliations:** ^1^School of Education, Central China Normal University, Wuhan, China; ^2^School of Psychology, Central China Normal University, Wuhan, China; ^3^School of Humanities, Hubei University of Chinese Medicine, Wuhan, China

**Keywords:** university–school collaboration, shared object, teacher development, teaching research activity, Chinese context, cultural–historical activity theory, contradiction analysis

## Abstract

**Introduction:**

University-school (U–S) collaboration has proven to be an effective approach for teacher professional development, but it could be hampered by the lack of shared objects. To understand how shared objects are formed in U–S collaboration, this research established a university-school collaborated Change Laboratory in W primary school based on cultural-historical activity theory, which is under the background of Chinese teaching research activity.

**Methods:**

Recordings of meetings throughout the year were transcribed into texts and coded, and then analyzed via the method of grounded theory and contradiction analysis.

**Results:**

The findings reveal that, in comparison to previous studies regarding shared object formation process, this study identified an special phase named “experimental object,” which highlights the significance of experimentation in U–S collaboration. Also, multiple contradictions are recognized as the driving force for shared object formation which would gradually transform into fundamental conflicts between tools. The main contradictions identified include those between scientific and daily concepts, university culture and school culture, as well as new experiment and old routine.

**Discussion:**

The current study implicates that U–S collaboration is an expansive learning process to acquire unknown knowledge, which necessitates both parties engaging in exploration and experimentation together. Furthermore, shared object formation within U–S collaboration requires participants to focus on developing teaching tools while consciously undergoing changes in aspects such as logic of thinking, culture and routine.

## 1 Introduction

### 1.1 University–school collaboration

In the past 20 years, with the aim of improving teacher education quality, primary and secondary schools in various regions have established collaborations with universities. This has proven to be an effective approach to teachers' professional development and students' learning outcomes (Moyer-Packenham et al., 2009; Jakhelln and Postholm, 2022). As the name suggests, university–school (U–S) collaboration refers to the collaboration between universities and schools for certain transform objects, which first emerged in Western countries. U–S collaboration is an outgrowth of the teacher professional development movement as a way to train in-service teachers and future teachers. According to Sirotnik and Goodlad (1988), to transform into model schools, schools must constantly receive new ideas and knowledge from the university, and the same applies to teachers. U–S collaboration not only promotes teachers' professional development but also allows university researchers to apply theoretical knowledge into practice. Furthermore, OECD (2015) advocates that U–S partnerships are central to fostering innovative teaching and learning–communities whereby a bridge is established between theory and practice and between practitioners and those engaged in academic research. Consequently, there is a growing trend toward establishing robust university–school partnerships to improve the quality of teacher education and promote student learning outcomes (Brady, 2002; Jakhelln and Postholm, 2022).

However, U–S collaboration faces many challenges in the complex socio-cultural context, such as value conflict, discourse power conflict (Jin and Lin, 2006; Zeichner et al., 2015), large cultural differences (Yang, 2011), conflict of forms and types of knowledge (Rachel, 2023), and the blurring of roles and responsibilities (Harford and O'Doherty, 2016). Among these challenges, the lack of shared objects between university researchers and school teachers is one of the major factors and prominent problems hindering successful U–S collaboration (Teng, 2008; Yamagata-Lynch and Haudenschild, 2009). Rachel (2023) has provided evidence to establish that working to find shared goals is vital if universities are really to work with schools in an equal way. Similarly, Halvorsen (2014) also notes that the ingredients of an effective partnership involve protecting each partner's identity while at the same time having shared goals.

The shared object is the premise of the U–S collaboration. Engeström et al. (1991) defined “collaboration” as the understanding and resolution of shared objects by organization members in a manner accepted by the public. In other words, a shared object is the foundation of collaboration. If people were all cognizant of the common end and interested in it so that they regulated their specific activity in view of it, then they would form a community that involved communication (Dewey, 2015). Also, the premise of collaboration is that both parties share common responsibilities, objects, and ideas (Teng, 2008). There is a discontinuity between university and school, which reflects the complex cultural contexts, and this affects the formation of common purposes. To promote the effectiveness of U–S collaboration, it is necessary to understand the differences between universities and schools so that both can interact, cooperate, and gradually understand each other to form common goals and beliefs in collaboration (Zhang, 2008).

### 1.2 Cultural–historical activity theory framework

Cultural–historical activity theory (CHAT) is applied around the world in various disciplines and domains of practice including in educational research. It is most commonly used as a conceptual lens through which data are interpreted (Gedera and Williams, 2016). The collective, transformative human activity system is often multi-voiced and multi-layered in that the actors have different roles, positions, and perspectives (Foot, 2014). CHAT uses a systematic analytical approach to uncover the varying and complex forms of human practices, both at the individual and social levels (O'Donoghue and Harford, 2020). CHAT considers human activity as a collective, artifact-mediated, culture-mediated, multi-voiced, and object-oriented activity system with six interconnected components, namely subject, object, tool, community, rule, and division of labor (Engeström, 2000, 2001, 2008).

**Figure 1:**
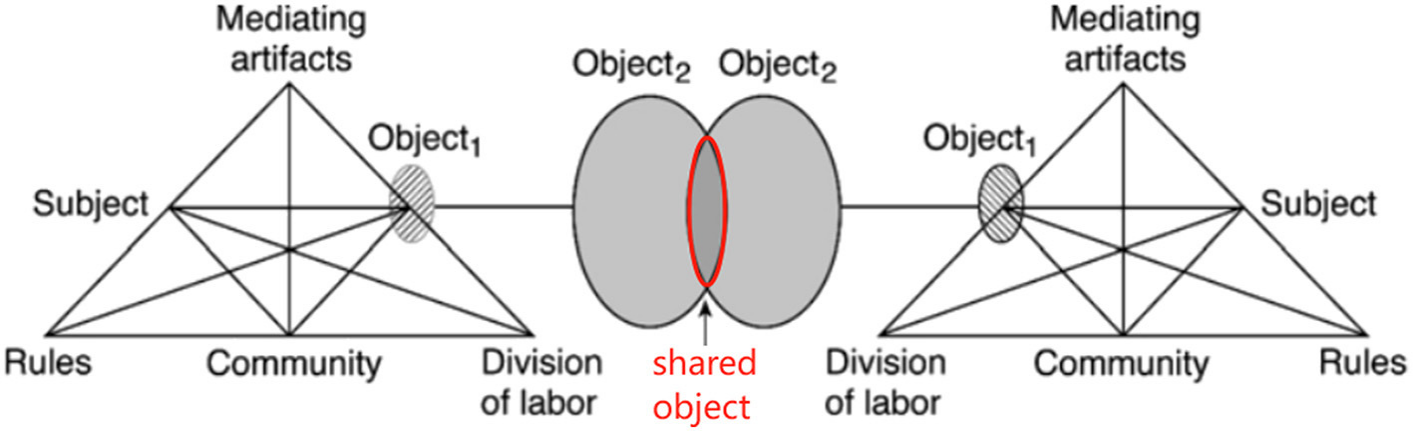
The third generation of CHAT (reproduced with permission from Engeström, 2001).

CHAT is thought to have evolved over three generations. The first generation of CHAT was proposed by Vygotsky (1978), who created the idea of mediation and a famous triangular model in which the mediating artifacts act as the conditioned direct connection between subject and object in human activities. The limitation of the first generation was that the unit of analysis remained individually focused. This was overcome by the second generation, centered around Leont'ev, wherein an activity system was regarded as the basic analytical unit of human activities, including the six elements. The third generation of CHAT, developed by Engeström, expanded the basic model to include at least two interactive activity systems to understand dialogue, multiple perspectives, and networks of interacting activity systems, with objects shared or jointly constructed by two or more activity systems named shared objects (Engeström, 2001) (Figure 1).

There are two foundational concepts of CHAT involved in this study: object and contradiction.

In CHAT, human activity is understood as an object-oriented system. The object of the activity is regarded as a defining component without which the activity could not exist (Leont'ev, 1978). Objects give shape and direction to activities and determine the horizon of possible actions (Engeström, 1995), and organizations are built and maintained around partially shared, partially fragmented, and partially disputed objects (Engeström and Blackler, 2005). The object of the activity is understood not merely as a thing but as the carrier of motivation, direction, sense of activity, as something “toward which an act is directed” (Leont'ev, 1981, p. 49; Engeström, 1995). It also is the carrier of use value and exchange value (Baudrillard, 1996, p. 90). Objects first emerge as raw materials or problems to be shaped and worked on. They then gradually take the shape of products or outcomes (Vetoshkina et al., 2017), which are not limited to physical things but also include relatively stable “immaterial” entities such as songs or theories (Leont'ev, 1981, p. 49). Moreover, objects are always in the process of transition and transformation, rather than a fixed or given state (Engeström and Blackler, 2005).

Activities are driven by objects, which in turn are generated and transformed through activities (Vetoshkina et al., 2017). Rantavuori et al. (2016) identified the object formation process by analyzing a collaborative curriculum learning process among Finnish pre-service teachers, namely the initial diffuse object, transitional object, and consciously articulated “germ cell” object. Moreover, the process of object formation was found to be iterative and non-linear. In other words, “object formation does not follow the ideal-typical phases.” Rantavuori's finding was built on that of Engeström and Kärkkäinen. Engeström (2001) roughly described shared object formation into three phases, namely raw material, collectively meaningful state, and potentially shared and co-constructed object., Kärkkäinen (1999) also identified three phases—routinized and fragmented or diffuse object, consciously articulated and shared “germ cell” object, and expanded object.

Another core concept is contradiction, which is historically accumulated structural tensions within and between the activity systems. CHAT considers the activity system as a community of multiple points of view, traditions, and interests, and contradiction is the central role of the activity system and the source of change and development, which generates not only disturbances and conflicts but also innovative attempts to change the activity, demanding both translation and negotiation (Engeström, 2001). Activity theory claims that actions do not take place in a stable, perfectly balanced context. An activity system is in constant imbalance and development. Development takes place as an outcome of the resolution of internal contradictions in the activity system (Engeström, 1995). Moreover, according to CHAT, learning is also a process that can be understood through contradictions in the system, wherein contradictions act as the driving force to promote the formation of shared objects in a collaborative activity (Turner et al., 2017; Ell and Major, 2019).

It is generally believed that contradictions can be divided into four levels (Engeström, 2015, p. 71)—primary, secondary, tertiary, and quaternary (Table 1)—and have four types of discursive manifestations (Engeström and Sannino, 2011) (Table 2). The primary contradiction pervades all elements of activity systems where activities are open systems. When an activity system adopts a new element from the outside (for example, a new technology or a new object), it often leads to an aggravated secondary contradiction where some old element (for example, the rules or the division of labor) collides with the new one. The tertiary contradictions mean contradictions between the object/motive of the dominant form of the central activity and the object/motive of a culturally more advanced form of the central activity. The quaternary contradictions exist between the central activity and its neighboring activities (Engeström, 2001).

**Table 1:** Four levels of contradictions (reproduced with permission from Engeström, 2015, p. 71).

**Contradiction level**	**Description**
Level 1	Primary inner contradiction within each constituent component of the central activity.
Level 2	Secondary contradictions between the constituents of the central activity.
Level 3	Tertiary contradictions between the object/motive of the dominant form of the central activity and the object/motive of a culturally more advanced form of the central activity.
Level 4	Quaternary contradictions between the central activity and its neighboring activities.

**Table 2:** Discursive manifestations of contradictions (reproduced with permission from Engeström and Sannino, 2011).

**Manifestation**	**Features**	**Linguistic cues**
Double bind	Facing pressing and equally unacceptable alternatives in an activity system. Resolution: practical transformation (going beyond words).	“we”, “us”, “we must”, “we have to”; pressing rhetorical questions, expressions of helplessness, “let us do that”, and “we will make it.”
Critical conflict	Facing contradictory motives in social interaction, feeling violated or guilty. Resolution: finding a new personal sense and negotiating a new meaning.	Personal, emotional, moral account narrative structure, vivid metaphors “I now realize that[..]”.
Conflict	Arguing or criticizing. Resolution: finding a compromise, submitting to authority or majority.	“no”, “I disagree”, “this is not true”, “yes”, “this I can accept.”
Dilemma	Expression or exchange of incompatible evaluations. Resolution: denial, reformulation.	“on the one hand[...] on the other hand”; “yes, but”, “I didn't mean that”, “I actually meant.”

Activity systems are characterized by inner contradictions. The primary inner contradictions reflect the basic contradiction characteristic of the socio-economic formation as a whole (Engeström, 1990, p. 84). Primary contradiction is omnipresent, lurking at the bottom of every activity in capitalism (Rocha, 2020). While Marx's ideas suggest that a trademark of capitalist social formations is the way surplus is pumped out from living labor (Marx, 1909), activity theory posits that the dual nature of commodities (i.e., their use and exchange value) is the primary contradiction existent among all activities (Engeström, 2001). The object of an activity carries within it the foundational contradiction between the use value and the exchange value (Engeström, 2015, p. xvi). In capitalism, commodities, including human beings, are contradictory unities of use value and exchange value (Leont'ev, 1981, p. 254; Engeström, 2015, p. xxix). For instance, “The doctor who buys a practice in some little provincial place may be very seriously trying to reduce his fellow citizens' suffering from illness and may see his calling in just that. He must, however, want the number of the sick to increase because his life and practical opportunity to follow his calling depend on that.” (Leont'ev, 1981, p. 254–255). The reciprocal and all-sided dependence of individuals who are indifferent to one another forms their social connection, which is expressed in exchange value. The power that each individual exercises over the activity of others or over social wealth exists in him as the owner of exchange of values, and of money. The individual carries his social value, as well as his bond with society, in his pocket (Marx, 1973, p. 156–157).

### 1.3 Teaching research activities

The background and object of this study is a U–S cooperative teaching research activity (TRA) in W Primary School, which aims to improve the effectiveness of TRA.

To some extent, TRA is similar to the concepts of action research, teacher professional development groups, and teachers research, all of which aim at the professional development of teachers and involve them in a cycle of inquiry, reflection, and action (Lytle and Cochran-Smith, 1989). However, TRA in China is almost unique in the world and is different from any teachers' activities in other countries. First, although there is teaching research in Western countries, schools do not have a mechanism and system dedicated to the development of teaching. Chinese TRA, on the other hand, is a comprehensive, complex, closely coordinated, and mutually promoting teaching research system. Second, action research in Western countries is mainly the behavior of a few individuals, and the research objects are often limited to the scope of personal teaching or serve for personal academic development requirements. While in China, TRA is a kind of collective, school-wide, regional, and even national action. Third, in terms of the number of participants in China, all teachers in all schools have to participate in TRA, forming a large-scale team of about fourteen million people (Cheng, 2021).

TRA has been greatly developed in the 21st century. Since the new curriculum reform in China in 2001 aimed at promoting well-rounded education in the 21st century, teachers have been facing greater challenges. Teachers are important pillars of education reform; hence, teacher education is a vital part of basic education reform. In China, the *Action Plan for Revitalizing Teacher Education (2018–2022)* stipulates that after nearly 5 years of efforts, teachers' comprehensive quality, professional level, and innovative ability should be improved considerably. Moreover, to adapt to the complex and changing times and educational environment, teachers need to improve their research ability and become research-oriented, which requires them to urgently find an effective means for their professional development. According to the *Opinions on Strengthening and Improving the TRA of Basic Education in the New Era (2019)* issued by the Ministry of Education, China, TRA has played a crucial role in promoting curriculum reform, guiding teaching practice, promoting teacher development, and improved educational decision-making, and it can provide support to promote teachers' professional development and improve the quality of basic education (Ministry of Education of the People's Republic of China, 2019). In order to improve the effectiveness of TRA, many schools also choose the U–S cooperative model, also known as U–S cooperative TRA.

All in all, TRA is an activity conducted by teachers to solve real problems encountered in education and teaching or the perplexities encountered in the process of school development. TRA is an integral part of the Chinese school system. The TRA organization includes TRA groups in schools and local education departments. The forms of TRA include teachers preparing for teaching and research together, seminar teaching, open class (teachers listen to each other's classes), teaching competition, learning in the studio of famous teachers, lecture training, action research, etc. (Paine and Ma, 1993; Cheng, 2021). Research objects of TRA are mainly the curriculum and its implementation, including teaching content, purpose, means, teaching mode and its construction, teaching design and implementation, and teaching evaluation, enabling teachers to reflect on, discover, and solve problems encountered in educational practice (Han, 2007). Chinese TRA enables teachers to form a huge learning community that constantly explores the unknown, keeps teachers in a state of constant reflection and renewal, and promotes their professional development.

### 1.4 Research questions and assumptions

Research on “shared object” originated in European countries, which can be mainly classified into concept research (Kärkkäinen, 1999; Engeström, 2001) and formation process research (Kärkkäinen, 1999; Engeström, 2008; Rantavuori et al., 2016; Virkkunen and Newnham, 2013; Zheng et al., 2021). However, how to form a shared object in U–S collaboration has been rarely discussed. Considering shared object is the premise to enhance U–S collaboration, it is particularly important to analyze the process and conditions of shared object formation. To clarify these questions, the current study established a university–school collaborated Change Laboratory in W Primary School based on cultural–historical activity theory, against the background of Chinese U–S TRA. This study addressed the following three questions:

1) What phases are involved in the formation of the shared object in university and school collaboration?
2) What are the contradictions in these phases?
3) How do these contradictions contribute to the formation of the shared object?

## 2 Method

The research design employed in this study was a qualitative methodology. Based on activity theory, a Change Laboratory was established in W Primary School, which lasted about a whole year, aiming to promote the effectiveness of TRA. The change lab, a basic formative-intervened research model, has been widely used in northern Europe to intervene and guide learning method changes in the workplaces of various social organizations. To demonstrate the formation process of a shared object in U–S collaboration, recordings of U–S collaboration meetings were transcribed into texts and coded, and the method of grounded theory and contradiction analysis were used for data analysis.

This research was approved by the research ethics committee at the School of Education, Central China Normal University. All procedures performed in the study involving human participants were in accordance with the ethical standards of the institutional research committee, and written informed consent was obtained from each participant.

### 2.1 Participants

Participants were from W Primary School, which has three main characteristics: First, it is located in a suburb of Wuhan, China, with most students being the children of migrant workers who are not highly educated and have less time and resources to spend on their children's education compared with other parents. Therefore, teachers' educational work in this school is more challenging than in other schools. Second, although TRA has been conducted for many years in this school, it is still inefficient and complex. Third, the school had been committed to “learning–centered teaching” for several years through TRA, but the outcome was not satisfactory. Additionally, the school has a large proportion of young teachers (70%); since young teachers are more energetic and open than older teachers, the former replaced the latter, thus becoming the vanguard in the TRA reform.

Participants in the Laboratory included schoolteachers and university researchers. As for teachers, there were a total of ten outstanding young teachers selected by the school principal from teachers who volunteered to participate in this research. It included five Chinese language teachers, two math teachers, two English language teachers, and one physical education (PE) teacher. Most of them were new teachers with < 2 years of teaching experience; only one had been teaching for 6 years. All the teachers were female except for the PE teacher (Table 3).

**Table 3:** The basic information of schoolteacher participants.

**Teacher**	**Gender**	**Subject**	**Grade**	**Age**	**Length of teaching**	**Education background**
1	Female	Chinese	4	25–30	1–2	Bachelor
2	Female	Chinese	5	25–30	1–2	Master
3	Female	Chinese	4	25–30	1–2	Bachelor
4	Female	Chinese	5	25–30	1–2	Master
5	Female	Math	2	20–25	1–2	Bachelor
6	Female	English	5	25–30	6	Bachelor
7	Female	English	5	20–25	1–2	Bachelor
8	Male	PE	4	20–25	1–2	Bachelor
9	Female	Chinese	3	20–25	1–2	Bachelor
10	Female	Math	5	25–30	1–2	Master

Participants also included seven researchers from a university. One researcher served as a researcher and an intervener, and two researchers controlled the process of the Change Laboratory, while other researchers recorded and videotaped the meeting sessions.

### 2.2 Data collection

The data was collected from ten recorded U–S collaboration meetings throughout the year (Table 4). University researchers and schoolteachers were the main participants in these meetings, but sometimes it also included school administrators. In each meeting session, the participants discussed a specific theme under the guidance of the researchers, and the sessions usually lasted for approximately 1.5 h, which was similar to the focus interview, but everyone had equal opportunities to talk and communicate freely. All meetings were recorded and videotaped by the researchers.

**Table 4:** Overview of U–S meeting sessions.

	**Meeting themes**	**Duration**	**Participants**
1	Engage with the school participants.	1:30:00	University researchers, W schoolteachers, and school administrators
2	Restart and discuss the activity object.	1:28:14	
3	Understand the challenges and problems teachers were facing.	2:33:31	University researchers and W schoolteachers
4	Discuss the disintegrated object.	2:22:02	
5	Propose a micro-class method and discuss its feasibility.	1:46:10	
6	Preparation for the implementation of the micro-class method.	1:42:23	
7	The first test of the micro-class method.	2:07:25	
8	The second test of the micro-class method.	2:09:48	
9	Optimize the class observation scale.	2:05:04	University researchers, W schoolteachers, and school administrators
10	Summary and future plans.	0:52:36	

### 2.3 Data analysis framework

As a first step in the analysis, grounded theory methodology was employed to illustrate the process of shared object formation in U–S collaboration. The recordings of the ten meetings were converted into textual format and subjected to triple coding for the identification of objects. Firstly, open coding was utilized to break down the data into potentially meaningful segments and identify underlying concepts. Subsequently, axial coding was adopted to locate and link action interaction within a framework of sub-concepts that provided significance and facilitated an explanation of ongoing interactions and consequences. The final phase was selective coding, where categories were integrated, structured, and saturated to determine if any new properties, dimensions, or variations emerged during the coding process. Following the independent triple-coding, cross-checking, and discussion among authors, subsequent modifications were made accordingly (Corbin and Strauss, 2008) and 15 activity objects were ultimately discovered and categorized into four groups (Table 5).

**Table 5:** Activity objects in U–S collaboration.

**Select coding**	**Axial coding**	**Open coding**	**Sources**
1. Fragmented object	Object from an academic framework	1. “We could divide the object into six aspects according to the six elements of activity theory.” (Professor M)	Transcript 3
	No idea	2.“I have no idea.” (Jiao)	Transcript 3
	Objects from daily teaching	3. “It is needed to improve the self-learning guiding sheet” (Lee)	Transcript 3
		4. “PE teachers need some PE teaching method.” (Jiao)	Transcript 3
		5. “I wanna observe the teaching of excellent teachers.” (Wang)	Transcript 3
		6. “It will be good to conduct TRA in different grades.” (Chen)	Transcript 3
		7.“I wanna do shadow learning.” (Zhang)	Transcript 3
		8. “It is needed to solve problems of underachievers.” (Huan)	Transcript 3
2. Initial collective object	Prior object	9. “How about reforming the TRA form first since it will be much easier and effective?” (Professor M)	Transcript 4
	Preliminary consensual object	10. “We agreed to reform the TRA form.” (teachers)	Transcript 4
3. Experimental object	No idea	11. “About how to reform the TRA form, we have no idea, and feel confused and doubtful.” (Lee)	Transcript 5
	Innovative object	12. “How about trying the micro-class method for TRA?” (Professor M)	Transcript 5
	Doubting, undecided object that need to be tested	13. “It is necessary to test the effectiveness and feasibility of the new method first.” (Lee, Zhang, Wang, Huan)	Transcript 6
4. Institutionalized shared object	Consensual and institutionalized object	14. “We are willing to continue using and improve the micro-class method for TRA.” (Lee)	Transcript 10
		15. “Let's popularize the micro-class method for TRA in the whole school.” (Headmaster)	Transcript 10

As a subsequent step, the D-analysis protocol (Middleton, 2011) was employed to elucidate the emerging strands of learning, i.e., how participants gradually reached a consensus on a shared object. The D-analysis protocol, which falls under communicative analysis, focuses on emergent distinctions that make the difference for participants in learning to do multi-agency work. It comprises the following five elements: deixis, definition and delineation, deliberation, departure, and development. Deixis is the initial concept that involves identifying when there is some nomination or “pointing” to a particular issue in terms of drawing attention to a distinction that is then developed to make a difference in subsequent turns of interaction. Deixis is then followed up by definition and delineation, where the issues are elaborated through qualifications, ordering, and expansive explanations provided by others involved in the discussion. Deliberation refers to the identification of how some working consensus emerges in terms of evoking both particularities and generalities of making distinctive features of past, present, or future practice. Departure involves identifying shifts toward qualitatively different positions in practices in terms of the formulation of emergent distinctions. Lastly, development refers to identifying when participants specify new ways of working that provide the basis for becoming part of or have become part of what they take to be and warrant a significant reformulation of their practices.

As a final step, to analyze the conditions of shared objects formation, we examined the contradictions within and between the university system and the school system based on the framework of four levels and discursive manifestations of contradictions developed by Engeström and Sannino (2011) and Engeström (2015) (Tables 1, 2). The theoretical basis of contradiction analysis is detailed and elaborated in Chapter 1.2 and will be further elaborated in Section 3.

## 3 Results

The activity objects underwent constant changes in both university and school systems. As shown in Table 5, a total of 15 objects were identified and divided into four groups. This indicates that the shared object formation in Chinese U–S collaboration involves four phases, namely specifically fragmented object, initial collective object, experimental object, and institutionalized shared object.

### 3.1 Phase 1–2: from fragmented object to initial collective object

In Phase 1, the object was fragmented, which originated from various understandings of the activity object of different subjects. For example, the professor proposed six disintegrated objects according to the elements of the activity system, while some teachers hoped to improve the self-learning guiding sheet, and some asked for the opportunity to observe teaching modes in other schools, some hoped the TRA would be conducted by grade. By identifying the essence of everybody's concerns and finding the common points, so-called “seeking common ground while reserving differences”, a consensus could be gradually reached. As a result, in Phase 2, the fragmented object was turned into the initial collective object (to reform the TRA form), which indicated that different subjects had reached a consensus on the activity object and could work together.

At the beginning of the third U–S meeting, participants began discussing how to improve the effect of TRA (deixis). Then, following the researchers' and some teachers' delineation, Professor M proposed six disintegrated objects of TRA reformation, which corresponded to the six elements of the activity system. For example, to foster teachers' enthusiasm (subject), to reconstruct teachers' community (community), and to change the TRA form (tool). However, the teachers maintained silence for several minutes, which probably indicated that they neither understood nor supported the proposal. It may be because the forms and types of knowledge espoused at the university level and at the school level are often antithetical, with universities typically promoting theoretical knowledge and schools focused on practitioner knowledge (Rachel, 2023). Later, in the fourth U–S meeting, Professor M asked teachers about their main concern about TRA. In response to this question, teachers immediately mentioned many problems faced in daily teaching, such as “lack of an imitable teaching method for a specific subject,” “lack of an opportunity of attending regional and other schools' training,” “difficulty for teachers from different grades to research together,” and “difficulty of cultivating students' independent ability.” (Excerpt 1). Obviously, the problems mentioned by the professor and teachers differed only in terms of expression and theoretical level; however, the contents seemed similar. For example, teachers also proposed “tool-regulated” problems (problem 1), “rule-regulated” problems (problem 3), and “object-regulated” problems (problem 4). Gradually, following sustained deliberations, the teachers began improving at the theoretical level and gaining a more comprehensive understanding of the practical problems, whereas the professor began to understand that teachers were concerned mainly regarding the form of TRA. Therefore, Professor M made some changes in the expression and viewpoints. Finally, a consensus on the object, that is, to give priority to reforming the TRA form, was reached. The sequence moved quickly from deliberation to departure and development, eventually forming the “initial collective object” to reform the TRA form, which would be considered the main topic in subsequent meetings.

**Figure 2:**
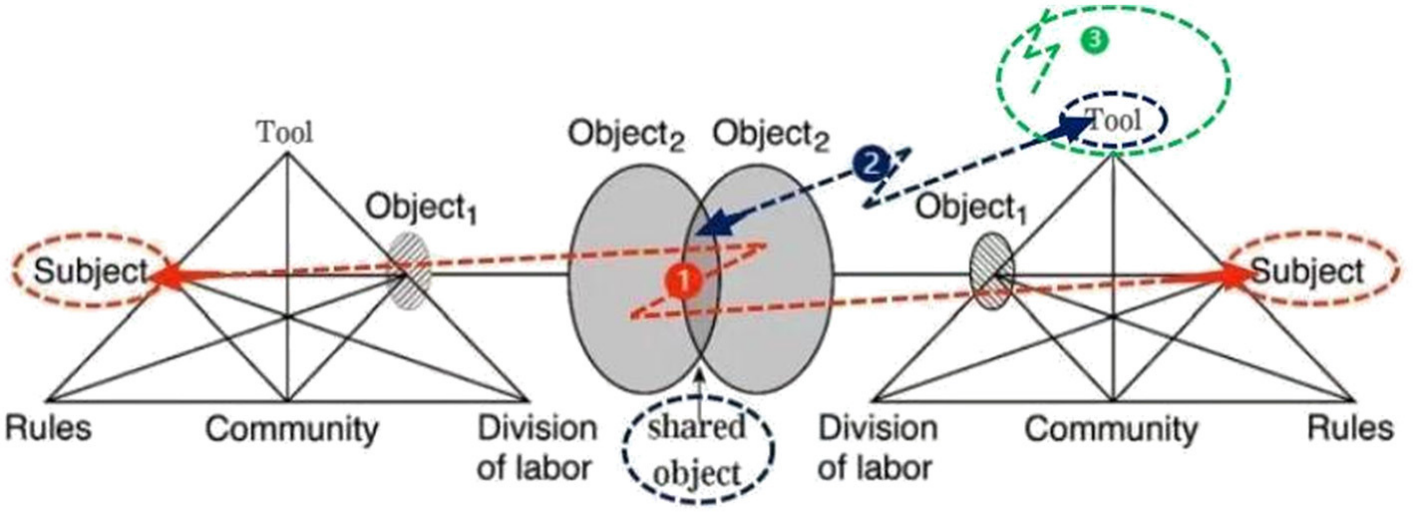
Contradictions in shared object formation process.

In Phase 1, the quaternary contradiction existed between subjects of the university system and the school system (Table 6; Figure 2), which was manifested by “critical conflict”. Professor M used the activity theory to guide TRA reform, but teachers did not understand his idea and were more concerned about the problems of daily teaching. Evidently, this reflected a disconnect from the school reality on the part of the academic world.

**Table 6:** Contradictions in shared object formation in U–S collaboration.

**Phases**	**Contradictions level**	**Contradictions content**	**Manifestation**	**Linguistic cue**
Phase 1–2	Quaternary contradiction	Contradiction between the subjects of two systems	Critical conflict	Jiao: (Teachers had been silent for a long time) I don't understand the integrated activity objects Professor M proposed. What concerns me most is...(Transcript A3&A4)
Phase 2–3	Secondary contradiction	Contradiction between the shared object and the tool of the school system	Dilemma	Wang: I know we have to change our TRA form, but I have no idea where to go; maybe we need help (Transcript A4).
Phase 3–4	Primary inner contradiction	Contradiction within the tools of the school system	Dilemma	Lee: I agree with the effect of the micro-class method for TRA. However, since we have gotten used to the old routine for a long time, and considering the limited time, I think there are still some difficulties in using the new TRA method (Transcript A10).

The essence of the quaternary contradiction was the contradictions between the scientific concepts of university researchers and the daily concepts of schoolteachers. Vygotsky (1978) proposed that human understanding could be influenced by two conceptual systems, namely scientific concepts and daily concepts. Scientific concepts are the systematic and theoretical ideologies derived mainly from books, whereas daily concepts are generated in authentic practical daily activity. Instead of being merely an academic term, the “concept” represents the understanding, idea, and attitude toward everything (Mao and Cai, 2012). What participants should do first with such a general goal as “TRA reform” depended on their understanding of the contradictions that are derived partly from scientific concepts and partly from daily concepts. In this study, the university professor had a profound theoretical foundation; however, his highly theoretical, systematic, and forward-looking scientific concepts were difficult for teachers to understand. Though the problems of daily teaching mentioned by teachers represented their daily concepts and rich practical experience, they failed to realize the essence of the problems. In this process, the university and school are disconnected. Fortunately, in the subsequent discussions, both sides tried their best to understand the essence of the issues expressed by the other, seek common ground while reserving differences, and then disintegrate the macroscopic object with their understanding to make it more operable. Gradually, the university professor understood the teachers' main concerns, and teachers, upon realizing the essence of the problem, improved at the theoretical level. At the end of the fourth U–S meeting, the subjects of the university system and the school system reached a consensus on the prior object of TRA reform.

**Excerpt 1:** (meetings 3 and 4)

*Professor M:* The headmaster entrusted us to make changes to the school. One purpose is to help improve our teaching research activities so as to improve your teaching efficiency and effectiveness. The other is to implement the school's learning-centered teaching mode. To carry out TRA reform, I suggest we start from six major aspects corresponding to the activity system's six elements oriented from CHAT theory. What do you think?

(silence)

*Professor M:* OK, let's move to another easier topic. What kind of support do you hope the school will provide for your future development?

*Wang:* Could the school organize us to learn teaching methods from other districts?

*Jiao:* For sports classes, since the older teachers' teaching methods are old-fashioned, there is little chance for young teachers to learn new good teaching methods.

*Zhang:* I want to take part in paired learning and learn from an experienced teacher throughout the day for a week.

(Part of the discussion is omitted here)

*Professor M:* Now I know your concern. Based on your problems and needs, our prior object may be to reform the TRA form.

### 3.2 Phase 2–3: from initial collective object to experimental object

Phase 2 involved merely a theoretical design; however, to verify whether a theory is correct, a practical test is essential. Thus, in Phase 3, the initial collective object was turned into the experimental object. The initial collective object refers to an object that is generally designed but not operable, whereas an experimental object refers to a specific object to be tested. For example, “to reform the TRA form” was the initial collective object, whereas “to test the micro-class method for TRA” was the experimental object.

At the beginning of the fifth U–S meeting, although teachers were aware of the need to change the TRA form, they lacked guidance and showed a great need for help (deixis). Later, the professor encouraged teachers to create an efficient TRA form through brainstorming, learning from advanced experience, and making innovative attempts. However, teachers admitted they did not know how to do it and were afraid of making attempts, asking experts repeatedly for directions and standard answers. Finally, out of respect and understanding for teachers' working culture, Professor M had to give up some principles and provided teachers with a new tool, that is, the micro-class method for TRA. Then, in the sixth U–S meeting, Professor M provided the definition, value, validity, and feasibility of the micro-class method. Micro-class refers to a 10–20-min class-teaching excerpt that can be used as a tool or a unit by teachers to explore the general principles and method of teaching using video and discuss the fragments of their classes. It is a novel tool and a new form of TRA generated in this research that can improve the effect and pertinence of TRA. To verify its operability, effectiveness, and feasibility, teachers needed to experiment independently. Immediately at the end of this meeting, deliberation on testing the new tool was formed. In other words, the sequence led to departure and development, and the four subsequent U–S meetings focused on the construction and testing of the new tool. The first step was the preliminary construction phase of the micro-class method, in which teachers exchanged ideas and shared their doubts. In the second step, the new tool was inspected. Teachers decided to apply the micro-class method to “cooperative learning” teaching activities and designed a measurement scale to better evaluate teachers' teaching behavior. After the first testing trial, teachers exchanged feedback and made some modifications to the micro-class method. Taken together, in this phase, the initial collective object was turned into the experimental object (to test the micro-class method for TRA).

In Phase 2, a secondary contradiction existed between the tool and the initial collective object, as the old TRA form (tool) could not meet teachers' new needs (object), and it was manifested by dilemma (Table 6; Figure 2). Teachers' new needs included an imitable teaching method, the opportunity of attending regional and other schools' training, conducting the TRA by grade, and instructing students for independent learning, whereas the old TRA form required teachers to gather to write teaching plans independently or engage in some administrative tasks. Overall, teachers needed a new form (tool) of TRA; however, they had no idea of what new tool could satisfy their new needs.

The essence of the secondary contradiction was the cultural contradictions between the university and the school. The core value of university culture is to create knowledge and thought, whereas that of school is based on practice, aimed at cultivating students by transmitting the wealth of human culture, which requires more attention to be paid to the application and transmission of knowledge (Yang, 2011). In the U–S collaboration, university professors hoped to guide teachers to give full play to their transformative agency and create a new TRA tool independently—as the professor said, “creating a new TRA tool requires you to brainstorm, learn from advanced experience, and make innovative attempts.” However, most teachers got accustomed to accepting what was provided directly by experts but lacked the ability to create new concepts or tools on their own—as a teacher said, “we have no idea of where to go; maybe we need your help, professor.” (Excerpt 2). Therefore, creating a new tool or form that can promote the TRA effect was difficult since it was important that all partners in a group were intrinsically involved in the planning, decision-making, implementation, and evaluation process (Gardner, 2011). After a long stalemate, struggle, and effort, each side finally adjusted themselves—the teachers decided to push themselves to create, and the professor also established a framework for the teachers. From the perspective of academic logic, teachers' professional development ultimately depends on their autonomy and creativity. However, from the perspective of practical logic, when teachers (especially in China) learn to do something new, they require certain referential experience to carry out reasonable innovations according to their situation (Chen, 2020).

**Excerpt 2:** (meetings 4 and 5)

*Professor M:* Creating a new TRA tool requires you to brainstorm, learn from advanced experience, and make innovative attempts.

(Part of the discussion is omitted here)

*Lee:* I know we have to change our TRA form, but we have no idea of where to go. Maybe we need your help, Professor.

*Professor M:..*.Okay...Since you have given your diligent efforts, I'm gonna help you. Let's think about the micro-class method, which can improve the effectiveness and pertinence of TRA. Micro-class means a kind of 10-20-minute class-teaching excerpt, also a tool that we can take as a unit to explore the general principles and method of teaching by video and discussion. What do you think of it?

*Zhang:* It sounds like that by using the micro-class method, we can focus on one point at a time. Different teachers teach and record the same class content so that we can compare and discuss their teaching.

*Xiao:* Using the micro-class method, the teaching process can be broken down into segments for discussion in a visual way.

*Lee:* The implementation of the micro-class method can effectively alleviate teachers' workload and enhance their efficiency. For instance, it enables efficient and effective exploration of specific topics while also providing a more authentic experience through self-recorded classes.

*Wang:* Not only do we lack a normative mechanism, but we also have technical problems; in addition, there has to consider the differences between disciplines.

*Professor M:* I understand what you are thinking about. All in all, whether the micro-class method for TRA suits us it needs to be tested. If we get a satisfactory result, then we can apply for approval from our school, and the micro-class method can be implemented in the future.

### 3.3 Phase 3–4: from experimental object to institutionalized shared object

In the last phase, the experimental object was turned into an institutionalized shared object. Although the experimental object (to test the micro-class method for TRA) was a type of collective object, it was still not institutionalized and was being tested, and before it passed several tests, all participants doubted its value, validity, and feasibility. The institutionalized object transformed from an experimental object was innovative and expansive, affirmed by all participants, and established as a new model or rule for daily activities. For example, establishing the micro-class method as a TRA tool in the whole school is an institutionalized shared object.

At the beginning of the tenth U–S meeting, participants were thinking about whether to continue using the micro-class method (deixis). During the preliminary modeling and testing phases, some teachers accepted the micro-class method, but some still had doubts. For example, some teachers felt that grasping its concept and procedure was challenging. This was followed by a presentation of the experimental result by teacher Lee, which delineated the concept, process, and effect of the micro-class method. She agreed with the TRA effect of the micro-class method and hoped to continue using and improving it; although she also expressed the difficulties faced by teachers “since we have got used to the old routine for a long time and still not familiar with the new method, and this new method is not yet mature, there are still some challenges in using the new tool”. (Excerpt 3). Deliberation was formed when the headmaster proposed that the new method was a good means for teachers' development and decided to implement it in the whole school. This also led to the departure and development that the new TRA method would be implemented by more teachers in the future, resulting in the institutionalization of the micro-class method as a new institution, new form, and a new tool of TRA. The schoolteachers proficiently began to use it in daily practice and explored means to combine it with various teaching contents. The micro-class method is expected to gradually improve in the long run. In this phase, the institutionalized shared object of the U–S collaboration had taken shape, implying that the micro-class method was established as an institutionalized tool in the whole school to improve the TRA effect.

In Phase 3, the primary inner contradiction, that is, the contradiction between the use value and the exchange value of tools, was evident in schoolteachers' activity system (Table 6; Figure 2), which was also manifested by “dilemma” as the contradiction between teachers' new experiment and old routine. The new TRA tool (micro-class method) had been improved greatly after two rounds of modeling and testing and was approved by most of the teachers. However, a few teachers who had been accustomed to the old TRA form considered it time-consuming and challenging as they were still not familiar with video lessons and the editing software. After discussions with participants and the headmaster, the micro-class method was then established as the institutionalized tool in the whole school to improve the TRA effect.

In this study, the micro-class method, as a new TRA tool, its use value lies in its ability to improve teachers' teaching effectiveness and research efficiency, and its exchange value is reflected in teachers' willingness to use it, that is, to what extent teachers are willing to invest time, energy, and change their past behavior habits to use this new tool. In fact, the previous TRA of teachers was completely formalistic. Teachers were required by the school to carry out TRA (“top-down” management approach), but they did not know the specific tasks and effective ways of carrying out TRA. At the same time, the school also required teachers to complete heavy teaching and administrative tasks. Therefore, teachers ultimately passively responded to the school's requirements in a formalistic way of collaboration, such as sitting together to complete their own lesson plans, work summaries, students' homework corrections, etc., but there was no actual collaboration, and they rarely discussed teaching issues. The new TRA tool (micro-class method) standardized the form of TRA. It was discussed in the Change Laboratory together by teachers and university researchers. The Change Laboratory, although organized by school administrators, was actively participated in by teacher representatives throughout the process. Teachers had full right to express and make decisions. In this sense, the emergence and implementation process of the new TRA form reflected the requirements and wishes of teachers rather than those of higher authorities or administrators. It is a combination of “bottom-up” and “top-down” management approaches. The micro-class method stipulates that teachers should focus on specific teaching issues in TRA and carry out discussions based on a pre-recorded micro-class video. The advantage of this form of TRA is that it is more targeted, focused, and efficient, which can promote genuine collaboration among teachers. Although the micro-class method may bring a learning burden to teachers in the short term, in the long run, it can improve teachers' teaching ability and research efficiency.

**Excerpt 3:** (meeting 10)

*Doctor D:* What do you think you have gained in this TRA reform? What do you think is good, and what needs to be improved? Everybody can say something.

(Part of the discussion is omitted here)

*Lee:* To some extent, we have seen the effect of the micro-class method for TRA. However, since we have gotten used to the old routine for a long time and are still not familiar with the new method, and this new method is not yet mature, I think there are still some challenges in using the new TRA method in a limited time.

*Headmaster:* Although there are still some difficulties, this new method is a good way for our teachers' development. For our TRA to be more efficient, we need to work together to overcome those difficulties. In the future, all teachers of the school will try out the new method.

To conclude, the contradictions of the shared object formation process in the U–S collaboration are presented in Table 6, which shows the linguistic cue, manifestation, content, and level of each contradiction in the U–S collaborated TRA.

## 4 Discussion and conclusion

### 4.1 The process of shared object formation in U–S collaboration

Compared with the three phases of shared object formation in previous studies (Kärkkäinen, 1999; Engeström, 2001; Rantavuori et al., 2016) (see Chapter 1.2), this study identified four phases in Chinese U**–**S collaboration, namely fragmented object, initial collective object, experimental object, and institutionalized shared object. In the first phase, the object is procedural, fragmented, and diffused, and it is derived from independent individuals or a single aspect. In the second phase, the collective object is initially established by two systems but lacks affirmation and acceptance by all participants. In the third phase, the object is tested to prove its value and improve itself. In the last phase, the shared object, which is innovative, expansive, institutionalized, and affirmed by all participants, is finally formed. It is worth noting that the process of shared object formation may not be linear. Although in this study, the shared object was formed step by step, in other cases, a certain stage may repeat itself several times; for example, the collective object may need to be reconstructed if the experiment doesn't work out, which was also proved by Rantavuori et al. (2016). Finding the special experimental object of the third phase in this study emphasizes the importance of the experiment in U–S collaboration. This phase is crucial because when the professor proposed the micro-class method, all participants doubted its feasibility, and some even did not agree with it and hence decided to first test it. The micro-class method was tested by teachers, and the theoretical design needs to be tested by practice. It was necessary to find out and solve the practical obstacles that teachers might encounter in the actual implementation process, which might cause some teachers to be reluctant to adopt micro-class method, such as teachers' unskilled use of educational technology, unwillingness to change their behavior habits or add teaching burden, and the low transformative agency, etc.

### 4.2 Contradictions of shared object formation in U–S collaboration

According to activity theory, multiple contradictions drive the formation of shared objects in a collaborative activity (Engeström, 2001). Our research not only supported this view but also found that with the shared object taking shape, the contradictions were gradually changing to the primary contradictions within tools. The first phase involved mainly the quaternary contradictions between the subjects of the university system and the school system, the second phase involved the secondary contradictions between the tool and the object, and the third phase involved the primary inner contradictions within the tools. Tools refer to the scaffolding that teachers use to improve their teaching (e.g., teaching patterns and TRA forms).

As analyzed in Chapter 3.3, in the third stage of U–S collaboration, the primary contradiction between the use value and exchange value of the new tool depends on teachers' willingness to adopt it. This willingness is contingent upon their personal transformative agency and learning ability (Diao et al., 2022), among which teachers' transformative agency is intricately linked to the approach of school transformation and management. This reflects the characteristics of the Chinese social culture and education system. The previous TRA form is the result of teachers' passive response to the “top-down” management approach of the school. Out of considerations of collectivism, Chinese teachers will comply with the school's management. However, the emergence and implementation of the new TRA form was the result of the joint action of teachers' transformative agency and school administrators' leadership, reflecting a combination of “bottom-up” and “bottom-up” school management approach, which led to teachers' relatively high transformative agency. However, although teachers have a certain transformative agency, they still need time to adapt to the new TRA form and change their old behavior habits. In addition, a few teachers who have insufficient transformative agency due to personal reasons still hold reserved attitudes toward the new TRA form. Finally, school administrators decided to implement the micro-class method throughout the school. In China, any rule established as an institution by the leader must be followed by everyone. Under the impetus of the institution, as if with a thrust, teachers' old habits were gradually replaced by new habits, and they gradually became accustomed to micro-class methods.

Since China's reform and opening up, the socialist market economy has both characteristics of autonomous, open, equal, competitive, socialist, collectivist, and state macro-controlled (Wu et al., 1993; Zhang, 1998). Chinese famous educational economists (Wang, 1994), as well as contemporary famous educators and Honorary President of the Education Association (Gu, 1993), have pointed out that this profound economic transformation has led to corresponding changes in all fields of social life, including education. Under the influence of the socialist market economy, China has formed an administrative management system combining centralization with decentralization (parallel implementation of top-down and bottom-up decision-making approach) (Gu, 1993; Zhang et al., 1993; Wang, 1994). In this study, the process of U–S collaboration in TRA reform and tool development also reflects this influence.

**Figure 3:**
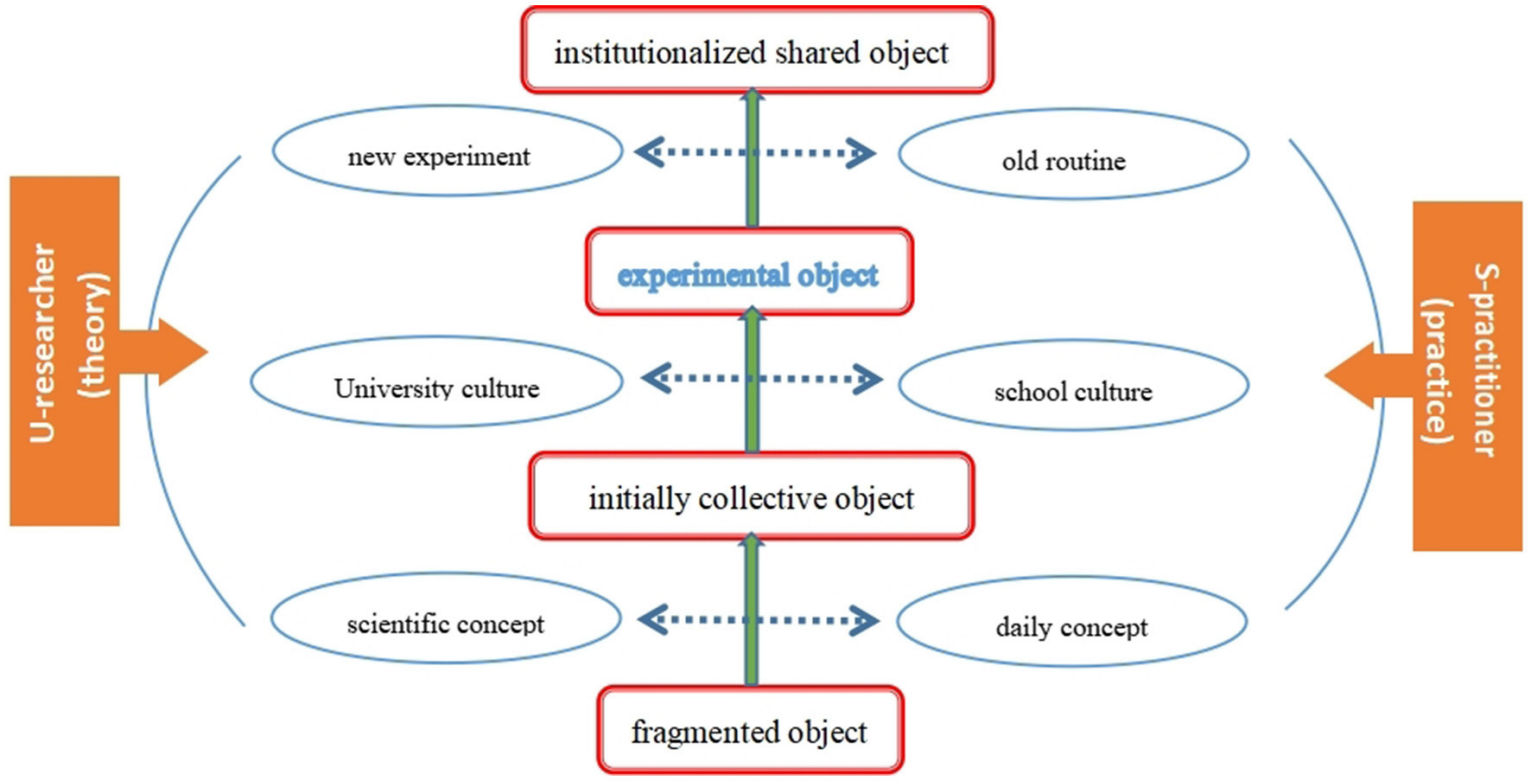
Essences of the contradictions in U–S collaboration.

In conclusion, the essences of contradictions identified in U–S collaboration are those between scientific concepts and daily concepts, university culture, and school culture, which will finally lead to contradictions between new experiments and the old routine of the school system. Fundamentally, the three pairs of contradictions in the U–S collaboration reflect the divided but interdependent relationship of theory and practice and the discontinuity between academics (universities) and reality (schools). University researchers mainly represent the theoretical and academic levels, whereas schoolteachers represent the practical and reality levels. Although the two are at different levels, there is a close relationship between them. The theoretical level encompasses scientific concepts, university culture, and new experiments, whereas the practical level includes daily concepts, school culture, and old routines. Each pair of elements corresponds to a pair of contradictions in the U–S collaboration (Figure 3). Scientific concepts and university culture together influence academic thinking and culture, while daily concepts and school culture together influence practical (reality) thinking and culture, which together lead to the discontinuity between academic (university) and reality (schools). This “theory-practice” divide is a perennial challenge in teacher education spaces (Harford and MacRuairc, 2008). Successful university-school partnerships explicitly question the binary of theory and practice (Flores, 2016, 2018) and encourage a positive attitude toward bridging universities' theoretical knowledge and teachers' practical knowledge (Nasri et al., 2023). La Velle (2019) argues that the theory-practice relationship should be seen not so much as a divide but as a nexus.

Both researchers and teachers understand that the close integration of theory and practice can improve the quality of teaching and learning, and teachers value the opportunity to gain research experience in collaboration with universities (Baumfield and Butterworth, 2007). As a result, in the process of shared object formation, each pair of contradictions interacts and changes the other pair until a state of equilibrium is reached, and both the scientific concepts of researchers and the daily concepts of teachers are changed in this process, leading to the formation of a consensus or a mature concept (Vygotsky, 1978). University culture and school culture also adjust to each other and, finally tend to be consistent. The same is true for new experiments and old routines. At this time, the shared object could be achieved.

### 4.3 Implication for successful U–S collaboration

This study has some implications for successful U–S collaboration. First, the experimental object in this study implicated that, in U–S collaboration, both sides usually unite to solve an unknown problem, which is a process of learning new knowledge rather than existing knowledge. Therefore, instead of being a process of moving directly toward a fixed object, its path represents a process of changing and adjusting the object, which is expansive learning (Engeström, 2001). University researchers are not authorities who know everything or provide a target for schoolteachers to follow but cooperate with them to explore, and it appears that a potential power dynamic may materialize between the teachers and university professors (Hamilton et al., 2021). This finding helps us better understand how the partners should position themselves since a key challenge in relation to understanding the nature of school-university partnership and of roles therein is achieving a clearer understanding of who participants are, how they understand their roles (Izadinia, 2014; Flores, 2016; Czerniawski et al., 2017) and making explicit the work they do in the collaboration (Flores, 2018).

Second, in successful and effective U–S collaboration, especially in U–S cooperated teaching research activities, both partners always focus on developing tools together, such as teaching models, teaching plans, and teaching strategies, which are required by teachers to improve their teaching. However, the process of tool development should adopt the “top-down and bottom-up combined” mode, which means that we should not only pay attention to the subjectivity of teachers and university experts but also emphasize the leadership of school administrators. In China, education policy drives education practice (top-down), which is the main way of education development. The top-down implementation method is conducive to the efficient formation of a unified guiding ideology and mobilizing the enthusiasm of all parties, but there are also some limitations. First, it is easy to ignore the scientific guidance at the practical level; second, in the process of “top-down” policy implementation, it will inevitably face “bottom-up” challenges, and it is easy to fail to understand the real needs and difficulties in practice. As a result, it is easy to lead to inadequate policy implementation, difficult to give play to the actual effect of policies, and even form a distorted implementation situation of “there are policies above and countermeasures below,” which runs counter to the original intention of policies (Beer et al., 1990, p. 68–69; Liu, 2016). The bottom-up implementation approach is conducive to giving play to the transformative agency of frontline educators, but they often only have scattered educational experiences, which makes it difficult to form systematic educational theories. Therefore, only by combining the two seemingly contradictory approaches of “top-down” and “bottom-up,” can educational development “use sufficient policy” and promote practice in a dynamic process (Fullan, 1994).

Third, to enhance U–S collaboration, it is necessary to focus on the differences between universities and schools so that they can interact, cooperate, and gradually understand each other, eventually forming common goals and beliefs in collaboration (Zhang, 2008), which requires frequent communication between members (Stevens et al., 1992). Not simply talking or engaging in conversation, communication involves the reframing of roles to engage from positions of hybridity, and both researchers and teachers adopt or incorporate the role of the other for greater interdependence and engagement (Pereira and Fang, 2022). Additionally, multiple contradictions were identified as the driving force to promote the formation of a shared object, to form a shared object in U–S collaboration; both sides should consciously change themselves in some aspects. Researchers should focus on changing their scientific concepts and work cultures, whereas teachers should focus on changing their daily concepts, work cultures, and routines.

### 4.4 Innovations and limitations

Based on CHAT, this research established a Change Laboratory in W primary school, which aimed to promote the effectiveness of TRA. To analyze the process and conditions of shared object formation in U–S collaboration, this study employed a qualitative research design with grounded theory methodology and contradiction analysis method. This research has the following innovations. First, it identified the process and conditions for shared object formation in U–S collaboration. Second, this study analyzed the contradictions between universities and schools in collaboration under the context of Chinese social culture. Third, the micro-class method as a new tool was established for Chinese TRA, which could improve the efficiency of TRA. Lastly, in terms of methodology, this study made some innovations to the “Change Laboratory” model in the Chinese context. For instance, Professor M in this study exhibited a dual identity as a researcher and an intervener, which is conducive to promoting communication between researchers and teachers.

However, there are some limitations to the study. First, due to the impact of the epidemic, we only had time to choose one school as the research sample. Second, although several researchers conducted multiple rounds of coding on the activity objects and reached a consensus with the authors, it is still difficult to avoid a certain degree of subjectivity.

## Data availability statement

The raw data supporting the conclusions of this article will be made available by the authors, without undue reservation.

## Ethics statement

This research was approved by the Research Ethics Committee at School of Education, Central China Normal University, Wuhan, China. All procedures performed in the study involving human participants were in accordance with the ethical standards of the institutional research committee. Written informed consent was obtained from each participant involved in the study.

## Author contributions

XF: Data curation, Formal analysis, Investigation, Methodology, Software, Visualization, Writing – original draft, Writing – review & editing. QM: Conceptualization, Funding acquisition, Methodology, Resources, Supervision, Validation, Writing – review & editing. JH: Methodology, Project administration, Resources, Supervision, Validation, Writing – review & editing. CD: Data curation, Formal analysis, Project administration, Software, Visualization, Writing – review & editing.
